# Disruption of Bcl-2 and Bcl-xL by viral proteins as a possible cause of cancer

**DOI:** 10.1186/1750-9378-9-44

**Published:** 2014-12-23

**Authors:** Kenneth Alibek, Stephanie Irving, Zarina Sautbayeva, Ainur Kakpenova, Aliya Bekmurzayeva, Yeldar Baiken, Nurgul Imangali, Madina Shaimerdenova, Damel Mektepbayeva, Arnat Balabiyev, Aizada Chinybayeva

**Affiliations:** Nazarbayev University Research and Innovation System (NURIS), Nazarbayev University, 53 Kabanbay Batyr Avenue, Astana, 010000 Kazakhstan; National Medical Holding, 2 Syganak Street, Astana, 010000 Kazakhstan; School of Science and Technology, Nazarbayev University, 53 Kabanbay Batyr Avenue, Astana, 010000 Kazakhstan

**Keywords:** Bcl-2, Bcl-xL, Herpesviruses, Human T-lymphotropic virus 1, Human papillomavirus, Hepatitis C virus, Apoptosis, Signaling pathways, Tumor suppressor genes, Cancer

## Abstract

The Bcl proteins play a critical role in apoptosis, as mutations in family members interfere with normal programmed cell death. Such events can cause cell transformation, potentially leading to cancer. Recent discoveries indicate that some viral proteins interfere with Bcl proteins either directly or indirectly; however, these data have not been systematically described. Some viruses encode proteins that reprogramme host cellular signalling pathways controlling cell differentiation, proliferation, genomic integrity, cell death, and immune system recognition. This review analyses and summarises the existing data and discusses how viral proteins interfere with normal pro- and anti-apoptotic functions of Bcl-2 and Bcl-xL. Particularly, this article focuses on how viral proteins, such as Herpesviruses, HTLV-1, HPV and HCV, block apoptosis and how accumulation of such interference predisposes cancer development. Finally, we discuss possible ways to prevent and treat cancers using a combination of traditional therapies and antiviral preparations that are effective against these viruses.

## Introduction

All cancers, together, comprise the second most prevalent cause of mortality worldwide. The development of strategies aimed at prevention and treatment to manage this disease critically depends on the understanding of cancerous cells and the mechanisms through which they arise [1]. It is becoming increasingly apparent that several viruses play significant roles in the multistage development of malignant cancers, where the correlation of a given virus with an associated cancer can range from 15-100% [1].

The human body naturally produces and destroys approximately 60 billion cells daily, and proper homeostasis is achieved by strict control of cell turnover [2]. Programmed cell death, or apoptosis, is an essential mechanism for regulation of tissue homeostasis, immune system functions, and embryo development, while abnormal and uncontrolled cell death is a major contributing factor to several diseases and tumorigenesis [3].

Bcl family proteins are key players in cell clearance. Therefore, when Bcl proteins are defective, it may lead to cancer initiation and promotion [3]. In fact, Bcl-2 was the first apoptotic regulator to be identified. In this case, the oncoprotein was activated via chromosome translocation (14:18 chromosome translocation) leading to human follicular lymphoma [3]. The role of the Bcl family in normal cells is to regulate apoptosis by inducing or inhibiting cell death according to environmental stimuli. Within the Bcl family, all anti-apoptotic Bcl-2 homologues function as oncoproteins, while pro-apoptotic and BH3-only proteins act as tumour suppressors.

Bcl proteins form homo- and heterodimers, which explains the neutrelising competition between these proteins. Each protein contains up to four conserved BH domains, which are alpha-helical segments that mediate interactions with other proteins [4]. The hydrophobic, C–terminal domain of these proteins localises them to intracellular membranes, such as the outer mitochondrial membrane, the endoplasmic reticulum, and the nuclear envelope [4]. It is hypothesised that anti-apoptotic proteins are initially integrated into membranes, while pro-apoptotic members are found in the cytosol. Only after receiving an apoptotic stimulus, pro-apoptotic Bcl proteins undergo a conformational change and are translocated into the mitochondrial membrane where anti-apoptotic proteins already reside [5–7].

**Figure 1:**
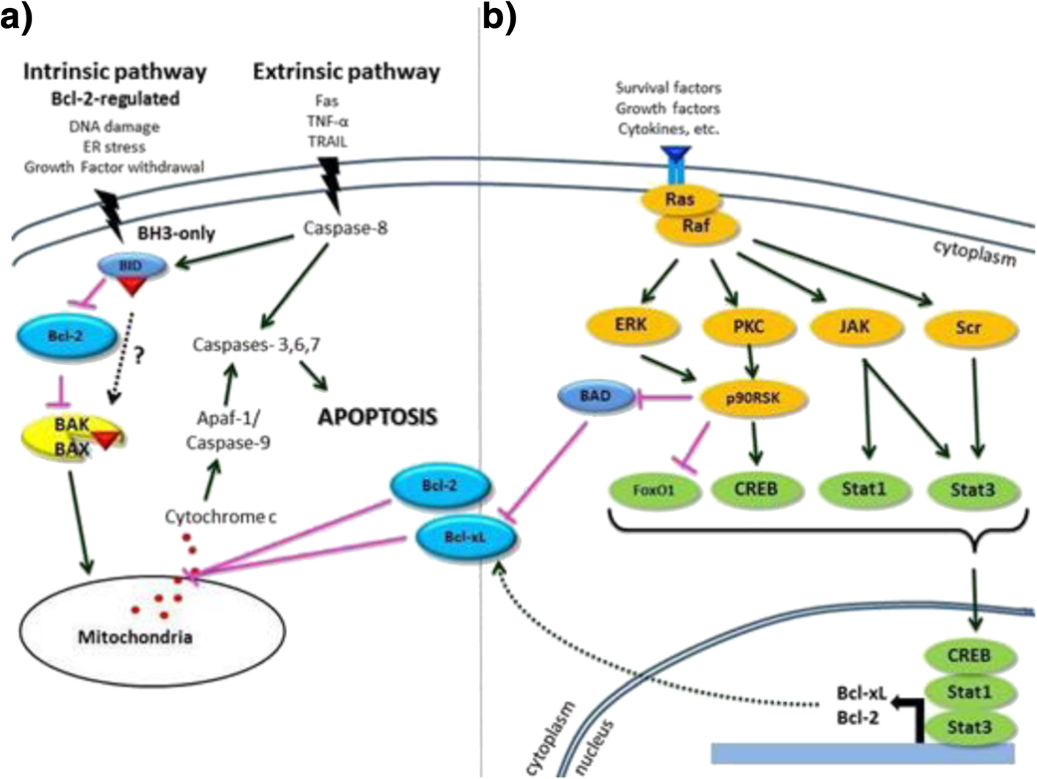
**(a) Apoptotic cell death pathways and (b) inhibition of the apoptosis signalling pathway.**

In total, the Bcl family includes more than 25 members in mammalian cells [8], which function as a ‘life/death switch’ that integrates diverse inter- and intracellular cues to determine whether or not the stress apoptosis pathway should be activated. The intrinsic apoptosis pathway is controlled by the Bcl family (Figure 1) [9]. In response to apoptotic stimuli, the balance and interactions of anti-apoptotic and pro-apoptotic Bcl proteins influence the activation of downstream pro-apoptotic proteins Bak (Bcl-2 homologous antagonist/killer) and Bax (Bcl-2-associated X protein) [10]. In an activated state, Bak and Bax change conformation and penetrate the mitochondrial outer membrane leading to cell death [11]. It is not fully understood how Bcl-2 regulates this mitochondrial pathway, except that Bcl-2 blocks the permeability of the mitochondrial outer membrane, preventing the release of cytochrome c and other pro-apoptotic proteins, which in turn activate proteases and caspases (Figure 1) [11, 12].

Upon viral infection, apoptosis plays a crucial role in host defense mechanisms. One of the more successful mechanisms used by viruses is the prevention of apoptosis in the virally-infected host cells. It is presumed that viruses have adapted the mechanism of inhibiting cell death in the host cell through Bcl-2 homologues, thus initiating their own survival in the host [13, 14]. We further analysed how viruses and their proteins interfere with the normal pro- and anti-apoptotic functions of Bcl-2 and Bcl-xL, propose possible treatment approaches to affect etiology of viral disease, which target pathological proteins and pathways, and discuss how this can be used in anti-cancer therapy.

### Epstein-Barr Virus (EBV)

Epstein-Barr virus (EBV) is a *gammaherpesvirus* carried by more than 90% of the world’s population, and it is associated with several lymphoid and epithelial malignancies, including Burkitt’s lymphoma, Hodgkin’s disease, and nasopharyngeal carcinoma [15] (Table 1). At least one homologue of the apoptotic inhibitor Bcl-2 is encoded by many gammaherpesviruses [16].

**Table 1:** **Viruses and their proteins that are related to possible cancer development**

Virus	Proteins and viral homologues	Related cancer type
Epstein-Barr virus	Latent phase: latent membrane protein 1 (LMP-1), LMP2A, EBV encoded small RNAs-1 (EBER-1), EBER2, BARF1, Lytic phase: BZLF1 andBHRF1	Lymphoid and epithelial malignancies including Burkitt’s lymphoma, Hodgkin’s disease, nasopharyngeal carcinoma, brain tumours, cervical cancer, leukaemia, gastric/stomach cancer, bladder cancer, breast cancer, colorectal cancer, renal cell carcinoma, and non-Hodgkin’s lymphomas
Human cytomegalovirus	IL-10, vMIA (viral mitochondria-located inhibitor of apoptosis), vICA (an inhibitor of caspase activation), c-FLIP proteins, pUL38, IE1(491a), IE2(579aa), US27, US28, UL33, and UL78	Brain tumour, breast cancer, cervical cancer, prostate cancer, colorectal cancer, Hodgkin’s lymphoma, lymphoma, nasopharyngeal cancer, Kaposi’s sarcoma, skin cancer, leukaemia, and bladder cancer.
Kaposi’s sarcoma-associatedherpesvirus	KSBcl-2 (encoded by KSHV ORF-16) and viral FLIP (FLICE inhibitory protein, ORFK13)	HIV-related cancers, Kaposi’s sarcoma, primary effusion lymphomas, Castleman’s disease, multiple myeloma, non-Hodgkin’s B-cell lymphoma, primary body cavity B-cell lymphoma, andbladder cancer.
Human papillomavirus	E6oncoprotein, E 7 oncoprotein, E5 oncoprotein	Cervical cancer, squamous cell carcinomas of head, neck, mouth, vaginal cancer, anal cancer, penile cancer, bladder cancer, vulva cancer, non-melanoma skin cancer, breast cancer, colorectal cancer, ovarian cancer, renal cell carcinoma, and pancreatic carcinoma.
Human T-cell leukemia virus	Tax, Rex, p12	Adult T-cell leukaemia/lymphoma and brain tumours (astrocytoma)
Hepatitis C virus	E1, E2, NS5A, KFBP38, BAX	HCC, liver cancer, B- and T-cell lymphomas, pancreatic cancer, hepatobiliary cancer, non-Hodgkin’s lymphoma, thyroid cancer

Previous studies have shown that EBV plays a crucial role in establishing its own latency and in tumour formation by interacting with host cellular factors, where the “factors” determine the fate of the host cell and influence the immune response to the infected host [15]. Interfering with multiple signalling pathways of the host cell enables EBV to escape the immune defense mechanisms of the host cell and resist apoptotic events to establish a life-long latent infection [15]. After EBV infection, in both latent and lytic phases, host cells express around 100 EBV proteins [15]. Some, such as latent membrane protein 1 (LMP-1), LMP2A, EBV encoded small RNAs-1 (EBER-1), EBER2, BARF1 (BamH1-A Reading Frame-1), BZLF1 (BamHI Z fragment leftward open reading frame 1) and BHRF1 (BamHI-H right reading frame 1), mimic the structure of the anti-apoptotic Bcl-2 proteins, and, thus, inhibit apoptosis of the infected host cell during replication [9].

BZLF1, expressed in an early stage of infection, inhibits MHC II-associated invariant chain (CD74) in CD4 T-cells, resulting in downregulation of Bcl-2 and Bcl-xL expression [15]. LMP-1 is essential for virus-induced B-cell immortalisation and protects B-lymphoma cell lines from apoptosis signals *in vitro* via induction of cellular Bcl-2 expression [17]. However, in epithelial cells, expression of high-levels of LMP-1 induces apoptosis, as there is no Bcl-2 expression to mediate the signal.

Bcl-2 has been shown to specifically block LMP-1-mediated apoptosis in LMP-1-containing cells in which Bcl-2 has been transfected. Moreover, co-expression of LMP-1 and Bcl-2 allows epithelial cells to grow under low-serum conditions [17]. Thus, Bcl-2 affects transformation of LMP-1 transfected epithelial cells in two ways: inhibition of apoptosis mediated by LMP-1 induction and interaction with LMP-1, which contributes to cell growth. Therefore, the process of EBV-associated epithelial cell transformation is likely regulated by co-expression of these two proteins. In fact, in malignant EBV-positive epithelial tumour and nasopharyngeal carcinoma cells, LMP-1 and Bcl-2 are frequently co-expressed [17].

The relationship between EBV and various malignancies is emphasised with post-transplantation lymphoproliferative disease (PTLD), as it is almost always associated with EBV infection [18]. Bcl-2 similarly confers resistance to apoptosis and has been implicated in the pathogenesis of a variety of malignancies, including lymphomas [18, 19], where Bcl-2 is expressed in the majority of lesions examined. Further, Bcl-2 was expressed in all but one LMP-1-positive case, and the lack of Bcl-2 expression is always associated with the absence of LMP-1 [18].

**Figure 2:**
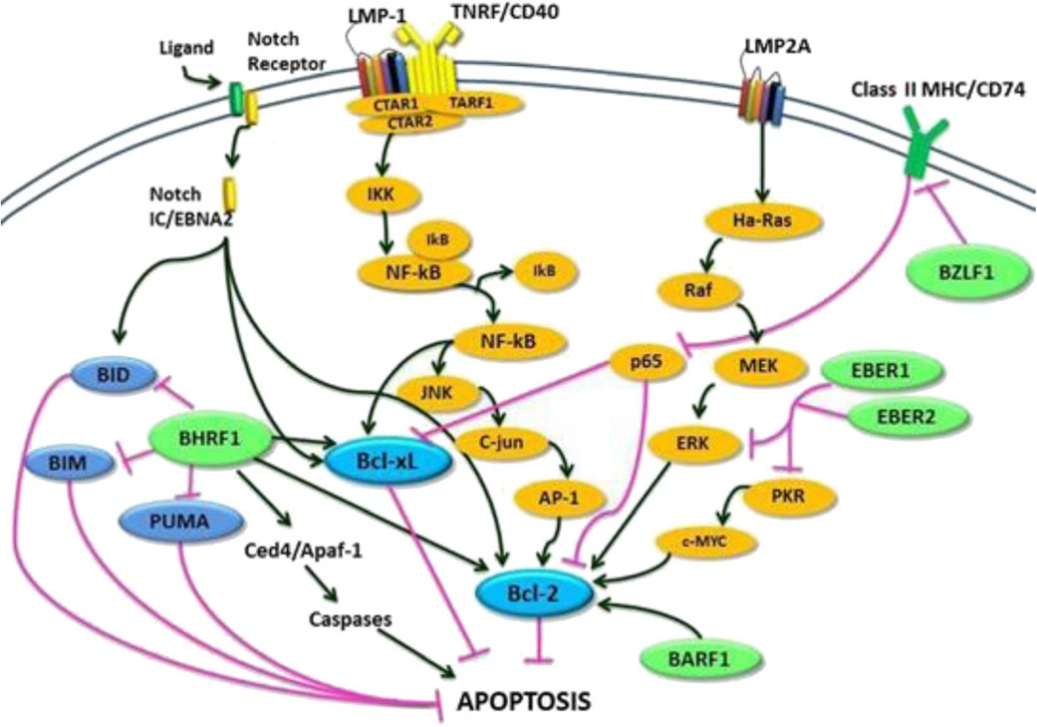
**EBV interaction with the Bcl family.** BHRF1 is the viral structural and functional homologue of the human proto-oncogene Bcl-2 and is able to blockcell death through repression of pro-apoptotic proteins BIM, PUMA, and BID and upregulation of Bcl-2 [15, 21]. Activated BARF1 upregulates Bcl-2 protein levels [15]. LMP-1 upregulates Bcl-2 expression through the NF-κB/c-JNK/AP-1 pathway, while LMP2A increases expression of Bcl-xL and Bcl-2 through the Ha-Ras, PI3K/Akt, NF-κB and Raf/MEK/ERK pathways, respectively [15]. BZLF1 represses CD74 and p65 resulting in downregulation of Bcl-2 and Bcl-xL expression [15]. EBER1 and EBER2 allow c-MYC to stimulate oncogenesis and inhibit apoptosis [15].

BHRF1 is a viral protein that is a structural and functional homologue. Bellows and colleagues determined that BHRF1 is an anti-apoptotic early lytic cycle protein, which is capable of preventing host cell death during viral lytic replication [20]. Both BHRF1 and Bcl-2 contain eight alpha helices and have a similar cellular distribution, being localised mainly in the nuclear membrane, endoplasmic reticulum, and mitochondria [9, 15]. Both lytic and latent BHRF1 transcripts were detected in EBV-positive B- and T-cell lymphomas. BHRF1 acts by changing its conformation and binding to and deactivating Bak. In cytokine-deprived cells, BHRF1 inhibits apoptosis by binding to BIM (Bcl-2 interacting mediator of cell death) instead of Bak. In addition, BHRF1 also represses pro-apoptotic BH3-only proteins PUMA (p53 upregulated modulator of apoptosis) and BID (BH3 interacting domain death agonist) and induces expression of host Bcl-2 and Bcl-xL genes [19]. The interactions between EBV proteins and Bcl-2 are shown in Figure 2.

### Human Cytomegalovirus (HCMV)

Human cytomegalovirus (HCMV), also known as human herpesvirus-5 (HHV-5), belongs to the *Herpesviridae* family. It is prevalent, with seropositivity varying from 50–90% of the population depending on socio-economic status. Upon infection of healthy individuals, the virus establishes itself in the haematopoietic cell population in the bone marrow and latently persists in the organism for a long period of time; however, it can be life-threatening for immunocompromised people and newborns. The virus has an oncomodulatory role and is frequently associated with breast, cervical, prostate, and colon cancers, brain tumours, and Hodgkin’s lymphoma [22].

The HCMV genome is 236 kbp and consists of immediate early, and late genes, which are crucial for the regulation of latent and lytic phases. Latency is maintained similarly to EBV and herpes simplex virus (HSV) and, under specific stimuli, the virus reactivates from latency to the lytic state. The first event is the expression of immediate early (IE) proteins from the IE locus, followed by expression of the early and late genes in a temporal cascade [23].

One of the main features of HCMV is its ability to inhibit apoptosis to allow additional time for further replication, leading to viral survival. HCMV encodes viral proteins that interfere with apoptotic pathways and prevent cell death. Ultimately, apoptotic events are deactivated because prosurvival proteins, such as Bcl-2, Bcl-xL or Bfl-1/A1, are highly overexpressed in virally infected cells (Figure 1) [24]. For example, viral gene UL37 exon 1 encodes the viral mitochondria-localised inhibitor of apoptosis (vMIA). This product directly binds Bax and blocks Bax-dependent pro-apoptotic activities using two mechanisms. The first is that vMIA sequesters Bax, inhibiting the process of outer mitochondrial membrane permeabilization. Second, vMIA recruits Bax to the mitochondria-associated membrane subcompartment of the endoplasmic reticulum, where it is actively degraded [25].

HCMV encodes special interfering proteins, such as vMIA, vICA (an inhibitor of caspase activation), functional homologues of Bcl-2, c-FLIP proteins, pUL38, IE1 (491a), and IE2 (579aa), which specifically inhibit caspase-dependent apoptosis, thus promoting survival of infected cells [26]. Experimental deletion of such genes from HCMV results in apoptosis. Fibroblasts at the G0-phase, when infected with high levels of HCMV, undergo less cell death and exhibit a 10-fold increase in Bcl-2 expression. When HCMV proteins IE72, pp65, and gB are expressed, markers of mitochondrial apoptosis (cytochrome c and caspase 3) accumulate in cells, indicating dysfunction of programmed cell death [27].

HCMV produces US27 and US28 proteins, which are G protein-coupled receptors (GPCRs) that play an important role in immunomodulation and virus persistence. For instance, the US28 gene product is a chemokine receptor that increases cell growth and can induce apoptosis in some cells. Furthermore, expression of US27 causes upregulation of the pro-survival factor Bcl-x, AP-1 transcription factor components jun and fos, and the IL-6 family cytokine oncostatin M (Figure 1) [28].

HCMV-infected cells interfere with NF-κB, which promotes a mesenchymal phenotype and metastatic spreading. The viral protein IE1 mediates the binding of NF-κB complexes to the *RELB* promoter, resulting in RelB synthesis, which then induces Bcl-2, leading to more invasive breast cancer cells [29]. Also, in HCMV-infected monocyte-derived macrophages, Bcl-3 is upregulated and associates with p52, which is a major component of NF-κB in cells [30]. Thus, apoptosis of HCMV-infected cells is affected by Bcl gene family products, and the cells acquire malignant phenotypes.

### Kaposi’s Sarcoma-Associated Herpesvirus (KSHV)

Kaposi’s sarcoma-associated herpesvirus (KSHV) belongs to the *Herpesviridae* family and is also known as human herpesvirus-8 (HHV-8). It is a *rhadinovirus* in the gammaherpesviruses subfamily. KSHV is strongly associated with HIV-related cancers, Kaposi’s sarcoma, primary effusion lymphomas, and multicentric Castleman’s disease [31–33]. KSHV is a large, double-stranded DNA virus with a genome containing a 140kbp unique coding region. This region is flanked by multiple GC-rich terminal repeat sequences and contains all of the KSHV genomic open reading frames (ORFs) [34]. There is a clear structural and biological similarity between KSHV and EBV; however, KSHV latency genes show no homology to EBV latency genes [35].

The life cycle of KSHV is biphasic with latent and lytic phases of replication with different genes being expressed in each phase. Therefore, like other herpesviruses, KSHV exhibits an initial lytic infection during which the linear viral genome is replicated, followed by lifelong latent infection, during which episomes containing the viral genome are produced [36]. Latency is essential for establishing persistent infection by suppressing apoptosis and for escaping the immune response of the host. Moreover, latent replication promotes tumour formation [37].

Many gammaherpesviruses, along with other herpesviruses, encode Bcl-2 with 20-30% homology between each other [16]. KSHV encodes a protein with both sequence and functional homology to human Bcl-2 [13, 38]. Moreover, KSHV Bcl-2 shows structural homology to Bcl-xL and Bax, two other members of Bcl family [39, 40]. KSHV Bcl-2 has a high degree of homology in the BH1 and BH2 domains with limited homology to other regions of Bcl-2 (BH3 and BH4). These conserved BH1 and BH2 motifs are necessary for the formation of homo- and heterodimers with other family members [38, 41]. The BH3 domain plays an anti-apoptotic role in KSHV Bcl-2; while, in other members of the Bcl-2 family this region functions as an apoptosis trigger. This difference is attributed to a missing non-structured loop between the BH3 and BH4 motifs in KSHV Bcl-2, which plays a role in the production of pro-apoptotic proteins [42]. The exact mechanism by which KSHV Bcl-2 prolongs cell survival is still unclear. Some studies assert that KSHV Bcl-2 does not form a heterodimer with pro-apoptotic Bcl-2 proteins. Instead, other studies reveal heterodimerization of KSHV Bcl-2 with anti-apoptotic Bcl-2 proteins [43, 44].

KSHV viral Bcl-2 (vBcl-2) is encoded by ORF16 [34]. This lytic protein acts directly on the apoptotic pathways via inhibition of apoptosis induced by KSHV infection and deactivation of the pro-apoptotic protein Bax [45, 46]. Introduction into yeast and human cells demonstrates that KSHV vBcl-2 suppresses Bax toxicity, and it heterodimerizes with human Bcl-2 (huBcl-2) in a yeast two-hybrid system [46, 47]. Expression of KSHV vBcl-2 prolongs cell life allowing the virus to replicate.

Another way to protect cells from apoptosis is to express KSHV viral cyclin (v-cyclin), encoded by ORF72. Unlike cellular Bcl-2 proteins, KSHV Bcl-2 is not a substrate for v-cyclin –CDK6 phosphorylation and does not contain a caspase cleavage site for production of pro-apoptotic proteins [48, 49]. Furthermore, viral FLIP (FLICE inhibitory protein, ORFK13), as well as vBcl-2 has anti-apoptotic properties, which might contribute to a poor prognosis in a subset of patients with Kaposi’s sarcoma [49].

Pro- and anti-apoptotic activity of Bcl-2 family members largely depends on mitochondria [50]. KSHV Bcl-2 inhibits the apoptotic signalling pathway by abrogating the function of mitochondria [13]. Repression of anti-apoptotic activity of KSHV Bcl-2 can be achieved by its translocation to the nucleoli via interaction with PICT-1. Nucleolar translocation shifts KSHV Bcl-2 away from mitochondria and, therefore, facilitates the downregulation of KSHV Bcl-2 anti-apoptotic activity [13, 51].

### Human Papillomavirus (HPV)

Human papillomavirus (HPV) is the most studied form of *papillomavirus*. There are over 100 different types dispersed among mammals, birds and reptiles [52]. HPV infects the epithelial cell layer of the mouth, anus, vagina, vulva, and cervix, as well as head, neck, and laryngeal mucosa. HPVs are classified into supergroups, where groups A and B contain the members most likely to lead to tumour formation [53, 54]. HPV 16/18, a member of supergroup A that has a genome of about 8000 base pairs, is considered a high-risk type of HPV that can cause cervical cancer [55]. Consisting of small double-stranded DNA, the HPV genome encodes six early viral proteins (E1, E2, E4, E5, E6, and E7) for DNA replication, and two late viral proteins (L1 and L2) for packaging of newly synthesised virions. The life cycle of most HPVs is a simple process where internalisation occurs via endocytosis of clathrin-coated vesicles [56]. After internalisation has occurred, viral proteins E1 and E2 are expressed to maintain the viral genome as an episome [57].

E6 and E7 are two proteins that play a crucial role in the life cycle of HPV 16. After internalisation into the host cell, these proteins are involved in cell proliferation by interaction with cell cycle regulators [58]. In particular, E7 interacts with the pRb pocket protein blocking the E2F transcription factor binding site, thereby promoting expression of proteins involved in DNA replication [59]. In addition, E7 can interfere with histone deacetylase and cyclin-dependent kinase inhibitors like p21 and p27, which strongly indicates the importance of E7 in proliferation of infected cells. On the other hand, E6 interacts with p53, leading to degradation via ubiquitin-dependent proteolysis [60–62].

Since p53 blocks expression of the anti-apoptotic Bcl-2 protein, E6 promotes increased Bcl-2 expression [63]. Thus, it is clear that E6 and E7 complement each other in promoting an anti-apoptotic effect. Moreover, E6 can act independently of E7 by stimulating the development of metastasis in cancer cells by its C-terminal PDZ-ligand domain, which leads to disruption of normal cell adhesion [64]. Other proteins such as E4 and E5, as well as E1 and E2, are involved in viral DNA replication [65].

Despite research on the HPV life cycle and the specific roles of viral proteins, the mechanism of the HPV life cycle is not fully understood. Although most HPV 16 is not directly involved in Bcl-2 activation, it is clear that there is a connection between E6 and Bcl-2 expression, which could play a direct role in cancer development.

Extensive investigation has revealed that the HPV 16 oncoprotein E5 plays a crucial role in driving cancerous mechanisms [66]. E5 is involved in the transformation of fibroblasts and keratinocytes as well as tumour progression of skin cancer in transgenic mice via epidermal growth factor receptor (EGFR) activation [67–69]. Further reports portray E5 as an additional helper to aid the E6/E7 triggered cancer development process [70]. Following infection, Bcl-2 family members display differential expression. Specifically pro-apoptotic Bak and Bax are decreased, and Bcl-2 levels increase [71]. Such an imbalance of apoptotic regulators creates a favourable environment for cancer development and progression. It is still unclear how each of the Bcl-2 family proteins interacts with the E5 oncoprotein; however, there is important evidence for the underlying mechanism of pro-apoptotic Bax protein regulation by E5 [71]. Bax is inhibited by E5 via stimulated ubiquitin–proteasome-dependent degradation, which involves EGFR activation and leads to the sequential reaction of cyclooxygenase-2 (Cox-2) upregulation. This results in vascular endothelial growth factor (VEGF) induction triggering angiogenesis, which is a critical step of cancer development and metastasis. In addition, Cox-2 induces Prostaglandin E_2_ (PGE2) which activates the expression of EP2 and EP4 (PGE2 receptors, G protein coupled receptor subtypes), cAMP and PKA, leading to Bax ubiquitination. Hence, E5 inhibits apoptosis by decreasing the amount of active Bax protein [71].

### Human T-cell leukemia virus-1

Human T-cell leukemia virus or HTLV-1 is a member of the *Retroviridae* family with a genome consisting of 9 kb dsRNA [72]. HTLV-1 was the first human retrovirus to be associated with cancer [73]. It is a causative agent of adult T-cell leukaemia/lymphoma (ATLL) [74, 75]. HTLV-1 is capable of transforming normal peripheral blood lymphocytes and animal cells *in vitro* and can cause tumours in transgenic mice [76, 77].

As with most other viruses, HTLV-1 has strategies to evade apoptosis to fight host cell defense mechanisms. Cells infected with this virus, as well as ATLL cells, demonstrate resistance to pro-apoptotic stimuli, such as gamma irradiation and DNA damage [78]. Unlike other viruses discussed in this review, HTLV-1 does not have cellular homologues of anti-apoptotic proteins [78, 79]. Instead, HTLV-1 expresses Tax (transactivator protein X), which has a potent ability to modulate both cellular and viral genes [75, 80–82]. Tax protects HTLV-1-infected cells from cell cycle arrest and apoptosis, which usually occurs in cells with mitotic checkpoint dysfunction or irreparable DNA damage [78]. Tax is one of the most studied proteins of HTLV-1 [78]. Together with its anti-apoptotic effect, Tax causes genome instability and mutations, deregulates cellular energy exchange, and is able to induce regulatory factors that activate the replication of T-cells, resulting in its uncontrolled replication [72, 83, 84].

Inhibition of apoptosis by expression of Bcl-2 proteins is a hallmark of haematopoietic malignancies and is commonly linked with resistance to therapy [85]. Proteins expressed by HTLV-1 modulate levels of Bcl-2 family members. Thus, host gene expression is induced by Tax via signalling pathways such as NF-κB and AKT [78]. All of these pathways somehow regulate proteins from the Bcl-2 family.

In addition, Tax activates anti-apoptotic genes and downregulates pro-apoptotic genes via activation of NF-κB, which induces expression of Bcl-xL and Bcl-2 [86, 87]. By inducing expression of Bcl-xL, Tax increases survival of cells infected with HTLV-1 and inhibits apoptotic signals, leading to leukaemogenesis. Bcl-2 and Bcl-xL are upregulated in cells infected with HTLV-1. Uncultured leukaemic cells also had increased expression of Bcl-xL and this was associated with the severity of the illness. Moreover, overexpression of Bcl-xL in ATLL cells may lead to chemoresistance [88–90]. By activating NF-κB, Tax can also mediate inhibition of CD95-mediated apoptosis through induction of c-FLIP. c-FLIP induction inhibits caspase-8, which is not able to truncate Bid, its direct substrate, and thus cannot induce cytochrome c release. Moreover, Tax increases Bcl-xL expression [91].

HTLV-1 positive T-cells express the anti-apoptotic protein Bfl1 while HTLV-1-negative cells do not, indicating that Bfl1 expression is Tax-dependent, although other viral proteins may be involved in Bfl1 induction. Tax induces Bfl1 expression via NF-κB (canonical and alternative) and AP-1 pathways. Furthermore, inhibition of Bfl1 and Bcl-xL (but not Bcl-2) leads to sensitisation of HTLV-1 infected T-cells, making these proteins crucial for T-cell survival [85].

Tax can potently repress pro-apoptotic Bax expression. This Tax-dependent downregulation of Bax leads to an altered ratio of Bcl-2 to Bax, which favours cell survival over apoptosis [92, 93], as the ratio of Bcl-2 to Bax is an important determinant of programmed cell death. p12, a small protein encoded by HTLV-1, activates IL-2 receptors and stimulates Jak1/3 which, in turn, activates STAT5. STAT5 induces expression of anti-apoptotic genes such as Bcl-xL [78] and downregulation of pro-apoptotic proteins, such as Bcl-2-antagonist/killer 1 (BAK1), and anti-apoptotic Bcl-2-associated athanogene 4 (BAG4) [94].

Tax modulates an anti-apoptotic effect through regulation of several Bcl-2 family members. It induces Bcl-xL, Bcl-2, and Bfl1, but suppresses Bax via the NF-kB pathway. Tax is important for protecting HTLV-1-infected cells from caspase-dependent apoptosis and progression of leukaemogenesis.

### Hepatitis C Virus (HCV)

Hepatitis C virus (HCV) is a single-stranded RNA virus of the *Hepacivirus* genus in the *Flaviviridae* family. It is the only positive-stranded RNA virus among the known human oncogenic viruses. It has an approximately 9.6 kb genome that contains an ORF, which encodes a 3000 amino acid poly protein precursor [1, 95]. HCV employs effective viral immune strategies to achieve a life-long infection. This persistent infection is strongly linked to hepatic sclerosis, hepatitis, cirrhosis, and hepatocellular carcinoma (HCC) [1], where elevated Bcl-2 expression in liver diseases can lead to the development of HCC [96]. HCV-associated cancers, particularly HCC, pose a major health concern, as it is the sixth most common cancer worldwide. Hepatocarcinogenesis results from dysregulation of the balance between cell death and proliferation. One such mechanism is overexpression of Bcl-xL in HCC cells, which reduces apoptosis [97].

Bcl-2 inhibits apoptosis and contributes to cell survival and resistance to cell damage. Bcl-2 family members regulate cell death and correlate with the progression and pathogenesis of cancers [96]. The molecular mechanism by which Bcl-2 expression leads to HCC in patients with chronic HCV infection remains unknown. However, it is clear that HCV activates Bcl-2 expression, which regulates the STAT3 signalling pathway, leading to regulation of gene expression during HCV infection [96]. Certainly, multiple functions of HCV proteins impact cell signalling, indicating that both host cell and viral factors play a role in HCC [1].

HCV infection leads to the activation of proteins involved in antiviral response, including interferons (INFs), interferon regulatory factors (IRFs), interferon stimulated genes (ISGs), signal transducers and activators of transcription (STATs), and NF-κB [1]. Recent findings have demonstrated that some HCCs are resistant to Fas-mediated apoptosis either directly through the expression of HCV proteins or indirectly through upregulation of Bcl-2 family members. Bcl-2 inhibits apoptosis either by preventing the release of cytochrome c or by interfering with the recruitment of pro-caspase 8 to Fas receptors [98]. Furthermore, HCV inhibits apoptosis at the mitochondrial level through augmentation of Bcl-xL expression, caused by inhibition of caspase-3 activation [98].

HCV encodes structural proteins, such as the core protein and E1 and E2 proteins that form a virus particle, as well as non-structural proteins that are expressed in hepatocyte cell lines [99]. Recent studies report that the HCV core protein may perturb apoptosis either by inhibition or induction of apoptosis. STAT3 is activated upon direct interaction with the core protein, resulting in upregulation of anti-apoptotic Bcl-xL, leading to excessive cell proliferation [100]. Moreover, Bcl-xL expression is exerted through the core-dependent kinase pathway, resulting in caspase-3 inhibition, which leads to inhibition of apoptotic cell death [101]. Core-dependent induction of apoptosis involves the Bcl-2 family member Bax, which is activated indirectly and is important for the mitochondrial apoptosis signalling pathway [102].

HCV’s non-structural protein 5A (NS5A) plays an important role in viral replication, survival and pathogenesis [103–105]. Recent studies reveal that NS5A employs various strategies to interfere with apoptotic cell death; however, the exact molecular mechanisms are yet to be identified [106]. NS5A interacts with FK506-binding protein 38 (FKBP38) and shows structural homology in the BH1, BH2 and BH3 domains of Bcl-2, thus transmitting the anti-apoptotic function of the Bcl-2 protein to the mitochondrial outer membrane [107, 108]. Moreover, NS5A interacts with Bax to inhibit its pro-apoptotic function in hepatocellular carcinoma [109].

Recent studies have shown that over-expression of SMAD3, a major TGF-β signalling transducer, reduces susceptibility to HCC by sensitising hepatocytes to apoptosis via Bcl-2 downregulation [97, 98]. However, elevated SMAD3 levels are found infrequently and are sometimes present in other forms of cancers, such as pancreatic cancer and colorectal cancer [98].

### A potential role for antivirals in cancer treatment

It is of great therapeutic importance to understand how viruses mimic host proteins to inhibit apoptosis. Several studies [110–112] indicate that overexpression of Bcl-2 and its close relatives is a major component of chemoresistance. For example, based on the National Cancer Institute (NCI) panel of 60 diverse cancer cell lines, Bcl-xL expression levels strongly correlate with resistance to most chemotherapeutic agents [113].

Regarding EBV, at least five latent viral genes must be expressed for еру-transformation of B-cells: EBNA-1, EBNA-2, EBNA-3a, EBNA-3c and LMP-1 [114], where LMP-1 most closely mimics a classical oncogene. Previous gene transfer research demonstrates that EBV-encoded LMP-1 mediates viral transformation and protects from cell death, in part by induction of the anti-apoptotic gene Bcl-2 [115]. Therefore, LMP-1 suppression is critical for maintaining cell growth and survival in EBV-immortalised cells.

When targeting LMP-1 with antisense oligodeoxynucleotides in EBV lymphoblastoid cells, the oligodeoxynucleotides targets codons one to five of the LMP-1 open-reading frames and suppresses the levels of LMP-1 in EBV-positive lymphoblastoid cell lines, inhibiting its function [116]. Furthermore, treatment with Bcl-2 antisense substances significantly delays development of fatal EBV-positive lymphoproliferative disease [117]. Inhibition of proliferation, decreased expression of Bcl-2, induction of apoptosis, and high sensitivity to chemotherapeutic agents are associated with LMP-1 suppression. These observations suggest that LMP-1 suppression in EBV-associated malignancy could be a therapeutic target for cancer treatment, and Bcl-2 antisense therapy may represent a novel anti-tumour treatment strategy.

HCMV is another potential target for cancer therapeutics. For instance, HCMV induces expression of Bcl-2 in neuroblastoma cells, resulting in apoptosis inhibition and chemoresistance. Antiviral medications, such as Ganciclovir, reverse these processes and are effective against cancer [118]. Ganciclovir specifically suppresses viral DNA polymerase activity, while another antiviral drug, Fomivirsen, is an antisense RNA formulation, which disrupts the functioning of the viral mRNA [119]. Unfortunately, HCMV develops resistance to antiviral therapy [120]. Since HCMV secretes a viral homologue of interleukin (IL)–10, which is detected in glioma stem cells [121], it is possible to adapt a therapeutic method based on targeting HCMV-secreted IL–10 to combat HCMV-related cancers [122].

Therapeutic methods such as radiation therapy, chemotherapy, and surgical excision are not efficient enough to combat KSHV tumours [123]. Perhaps targeting specific molecules that alter signalling pathways in tumour cells will be a more efficient therapeutic method. Nutlin-3a, murine double minute 2 (MDM2) inhibitor, destabilises the complex formed by the MDM2, p53, and KSHV latency-associated nuclear antigen (LANA), which triggers large-scale apoptosis in KSHV-induced primary effusion lymphoma (PEL) cells [124]. This method of reactivating the p53 pathway is a possible treatment option for KSHV-induced lymphomas. Another potential treatment strategy for KSHV patients is RNA interference. A short hairpin RNA delivered by lentiviral vectors acted on the KHSV genome in the murine PEL model. In particular, viral cyclin (vcyclin), vFLIP and LANA were targeted. As a result, PEL cell lines exhibited apoptosis and ascites development was inhibited [125].

The cells of dermal Kaposi’s sarcoma are associated with high levels of phosphorylated Akt, p70S6 kinase, endothelial growth factor (VEGF), and Flk–1/KDR (fetal liver kinase-1) protein [126]. As the immunosuppressive drug Sirolimus (*rapamycin*) specifically targets phosphorylated Akt and p70S6 kinase, it is an effective treatment for Dermal Kaposi’s sarcoma. After three months of treatment with Sirolimus, there was a total disappearance of dermal Kaposi’s sarcoma symptoms [126].

Over 90% of cervical cancers are HPV positive [127]. One strategy for reducing the prevalence of cervical cancer is to prevent HPV infections. Recent options for HPV prevention include vaccination. A quadrivalent HPV vaccine study has shown that the vaccine effectively protects women from high-risk vulvar, cervical, and vaginal lesions of HPV 6, 11, 16 and 18 for up to a 3 years post-vaccination [128]. A 4-year trial of a preventive human papillomavirus HPV16/18 vaccine adjuvanted with AS04 (consisting of aluminum hydroxide and 3-O-desacyl-4′-monophosphoryl lipid A) (Cervarix, GlaxoSmithKline) indicates up to 100% efficacy against cervical intraepithelial neoplasia grade 2+ (CIN2+) associated with HPV 16/18, although efficacy lessens as patient age increases [129].

Therapeutic vaccines of various structure and composition targeting E6 and E7 are also widely studied. These include viral/bacterial vector-based, protein and peptide-based, and RNA/DNA-based vaccines, which all show significant protection in mice [130]. Furthermore, when mice are treated with adenovirus vector co-transfected with HPV 16 E5 genome-based recombinant tumour vaccine, they display a reduction of cervical tumour progression via cytotoxic T-cell lymphocyte development [131]. As there are many mechanisms of E5 involvement, such as additional modulation of E6/E7 driven tumorigenesis and EGFR activation, these pathways could be targeted for E5 oncoprotein inhibition and interference. However, this approach has not yet yielded encouraging results, and further research should continue [130].

An alternative strategy is to treat HPV infection to block viral-induced cancer development. Although several approaches are possible, the most promising one involves siRNA treatment [127]. Furthermore, E6 mRNA degradation via specific siRNA was successful to reduce both cell growth and p53 accumulation in HPV-positive cells [127]. Finally, HPV-negative cells didn’t show similar results when treated with antiviral siRNAs, which reveals the specific silencing effect on HPV-infected cervical carcinoma cells [127].

Untargeted conventional chemotherapy is ineffective for ATLL treatment, especially in the acute form of the disease. Therefore, other treatment regimes that target either HTLV-1, Tax, or secondary genetic effects might be required to treat ATLL [132]. An extensive review of various targeted treatment regimens suggest a combination of arsenic, IFN and Zidovudine might be successful in ATLL treatment, as HTLV-1 load is decreased by Zidovudine, and Tax is degraded by arsenic/IFN [133].

Other potential ATLL treatment strategies include antivirals and other agents that can directly induce apoptosis by inhibiting Bcl-xL, Bcl2, or inducing Bax. For instance, Ritonavir, an inhibitor of HIV protease, has an antiproliferative effect on ATLL cells *in vitro*. Ritonavir phosphorylates NF-κB, inhibiting its activity as shown by downregulation of Bcl-xL and other anti-apoptotic proteins [134]. Diterpenoid oridonin from *Rabdosia rubescens* alsoinhibits NF-κB, thus inhibiting downstream antiapoptotic Bcl-2 proteins. In ATLL cells, diterpenoid oridonin downregulates Bcl-xL, but not Bcl-2 [135]. Capsaicin (main component of red pepper) also inhibits NF-kB, resulting in a change of Bcl-2/Bax ratio, inhibiting ATLL cell growth [136].

Certain derivative compounds are capable of inducing apoptosis in ATLL cells and cause fewer side effects than the compounds from which they were derived. For instance, EAPB0203, derived from imidazo [1,2a] quinoxaline, inhibitscIAP1 and Bcl-xL in malignant T-cells with and without the virus present, and does not have the proinflammatory effect of imiquimod. This compound causes a decrease in mitochondrial membrane potential, the release of cytochrome c, and caspase activation. The effect of EAPB0203 is reversed by treatment with caspase inhibitors [137]. Other drugs, used in combination with conventional drugs, are able to increase their cytotoxic effects and apoptosis induction. This effect is demonstrated when the small molecule ABT737 is used in combination with doxorubicin, vincristine, or etoposide. This promising drug for the treatment of ATLL interacts with the BH3 binding groove of Bcl-2, Bcl-xL, and Bcl-w. ABT737 induces apoptosis in the ATLL cells of mice, both *in vitro* and *in vivo*. These results could have important implications for ATLL treatment [90].

There has been major progress in treatments available for HCV-infected patients. Until recently, the go-to treatment was a combination of pegylated interferon and Ribavirin, which failed to eliminate HCV infection in a large proportion of patients and also produce multiple severe side effects [138].

TNF-related apoptosis-inducing ligand (TRAIL) induces apoptosis in various transformed cell lines, but not in normal tissue. HCC cells express TRAIL; however, over-activation of NF-κB and Bcl-xL in HCC cells may restrict TRAIL-mediated apoptosis [92]. This has led to recent interest in the development of new therapeutic approaches which would sensitise HCC cells to TRAIL-induced apoptosis [92]. After HCV infection, the expression of Bcl-xL and Bak fluctuate, and there is a significant difference in RNA expression levels of both Bcl-xL and Bcl-2 genes in HCC when compared to non-infected individuals. Higher levels (or lower levels) of expression is significantly associated with poorly differentiated tumours [93]. The Bak gene induces apoptosis in HCC cells, despite the presence of high levels of the anti-apoptotic Bcl-2 family members [93], indicating a possible therapeutic route for controlling apoptosis in HCV-infected patients.

Results are not consistent for the combination of interferon-α and Ribavirin; however, it is a more effective therapeutic method than interferon- α alone [139]. Treating with a combination of the HCV protease inhibitor VX −950 and pegylated interferon-α displays an antiviral effect; however, results remain inconsistent [140]. The development of sofosbuvir, a direct acting antiviral agent, shows potent activity against HCV and has further been shown to improve the rates of sustained virological response. Sofosbuvir a nucleotide analogue (NS5B polymerase inhibitor), is a potent drug with excellent tolerability and pan-genotypic activity with a high barrier to resistance with powerful antiviral activity against all HCV genotypes. Used in combination with ribavirin, with or without pegylated interferon, sofosbuvir can produce high SVR rates following 12–24 weeks of therapy, where there is no viral presence in the blood [141].

Suppression of HCV RNA polymerases by various nucleoside and non–nucleoside polymerase inhibitors can potentially have an antiviral impact [142]. This suggests that therapies that alter the life cycle of HCV infection, particularly those that target NS5B RNA-dependent RNA polymerase and NS3/4A serine proteases, are potential therapeutic agents for cancer treatment [143].

## Conclusion

The Bcl-2 family has emerged as a dominant regulator of apoptosis in cancer cells. The mitochondrial-mediated apoptosis pathway is regulated by anti-apoptotic and pro-apoptotic (Bad, Bax, and Bak) proteins. Defects in apoptosis signaling directly contribute to tumorigenesis; therefore, the Bcl proteins are very important players in apoptosis. By affecting Bcl proteins or their expression levels, viruses benefit in many ways, such as inhibiting normal programmed cell death in host cells, which propagates infection. In this review, we have outlined the mechanisms by which viral proteins exploit or mimic the Bcl-2 family. We have focused on how Herpesviruses, HTLV-1, HPV and HCV take advantage of various pathways to block apoptosis, predisposing cells for malignant cancer phenotype. To counteract this, we have proposed possible therapeutics against viral infection, which have the potential to eliminate Bcl-induced carcinogenesis. Finally, we speculate to what extent treating viral infections may advance cancer treatment.

## Abbreviations

Bak: Bcl-2 homologous antagonist/killer
Bax: Bcl-2-associated X protein
EBV: Epstein-Barr virus
LMP-1: Latent membrane protein 1
LMP-2A: Latent membrane protein 2A
EBER-1: EBV encoded small RNAs-1
EBER-2: EBV encoded small RNAs-2
BARF1: BamHI-A Reading Frame −1
BZLF1: BamHI Z fragment leftward open reading frame 1
BHRF1: BamHI-H right reading frame 1
PTLD: Post-transplantation lymphoproliferative disease
BIM: Bcl-2 interacting mediator of cell death
PUMA: p53 upregulated modulator of apoptosis
BID: BH3 interacting domain death agonist
HCMV: Human cytomegalovirus
HHV-5: Human herpesvirus-5
HSV: Herpes simplex virus
IE: Immediate early
vMIA: Viral mitochondria-localised inhibitor of apoptosis
GPCRs: G protein-coupled receptors
KSHV: Kaposi’s sarcoma-associated herpesvirus
HHV-8: Human herpesvirus-8
ORFs: Open reading frames
v-cyclin: Viral cyclin
HPV: Human papillomavirus
EGFR: Epidermal growth factor receptor
Cox-2: Cyclooxygenase-2
HTLV-1: Human T-cell leukaemia virus
ATLL: Adult T-cell leukaemia/lymphoma
Tax: Transactivator protein X
BAG4: Bcl-2-associated athanogene 4
HCV: Hepatitis C virus
HCC: Hepatocellular carcinoma
INFs: Interferons
IRFs: Interferon regulatory factors
ISGs: Interferon stimulated genes
STATs: Signal transducers and activators of transcription
STAT3: Signal transducer and activator of transcription 3
NS5A: Non-structural protein 5A
FKBP38: FK506-binding protein 38
MDM2: Murine double minute 2
LANA: Latency-associated nuclear antigen
PEL: Primary effusion lymphoma
VEGF: Endothelial growth factor
Foetal liver kinase-1: Flk–1/KDR
TRAIL: TNF-related apoptosis-inducing ligand.

## References

[1] McLaughlin-Druber ME, Munger L (2008). Viruses associated with human cancer. Biochem Biophisica Acta.

[2] Kirkin V, Joos S, Zornig M (2004). The role of Bcl-2 family members in tumorigenesis. Biochim Biophys Acta-Mol Cell Res.

[3] Reed JC (1998). Bcl-2 family proteins. Oncogene.

[4] Gross A (2001). BCL-2 proteins: regulators of the mitochondrial apoptotic program. IUBMB Life.

[5] Hockenbery D, Nunez G, Milliman C, Schreiber RD, Korsmeyer SJ (1990). BCL-2 Is an inner mitochondrial-membrane protein that blocks programmed cell-death. Nature.

[6] Hsu YT, Youle RJ (1997). Nonionic detergents induce dimerization among members of the Bcl-2 family. J Biol Chem.

[7] Gross A, McDonnell JM, Korsmeyer SJ (1999). BCL-2 family members and the mitochondria in apoptosis. Genes Dev.

[8] Cory S, Adams JM (2002). The BCL2 family: regulators of the cellular life-or-death switch. Nat Rev Cancer.

[9] Kvansakul M, Hinds MG (2013). Structural biology of the Bcl-2 family and its mimicry by viral proteins. Cell Death Dis.

[10] Nechushtan A, Smith CL, Lamensdorf I, Yoon SH, Youle RJ (2001). Bax and Bak coalesce into novel mitochondria-associated clusters during apoptosis. J Cell Biol.

[11] Dewson G, Kluck R (2010). Bcl-2 family-regulated apoptosis in health and disease. Cell Health Cytoskeleton.

[12] Newmeyer DD, Farschon DM, Reed JC (1994). Cell-free apoptosis in xenopus egg extracts inhibition by bcl-2 and requirement for an organelle fraction enriched in mitochondria. Cell.

[13] Flanagan AM, Letai A (2008). BH3 domains define selective inhibitory interactions with BHRF-1 and KSHV BCL-2. Cell Death Differ.

[14] Inhibition of Apoptosis Signaling Pathway: **Inhibition of Apoptosis Signaling Pathway.** http://www.cellsignal.com/contents/science-pathway-research-apoptosis/inhibition-ofapoptosis-signaling-pathway/pathways-apoptosis-inhibition

[15] Fu Q, He C, Mao ZR (2013). Epstein-Barr virus interactions with the Bcl-2 protein family and apoptosis in human tumor cells. J Zhejiang Univ-Sci B.

[16] Hardwick JM, Bellows DS (2003). Viral versus cellular BCL-2 proteins. Cell Death Differ.

[17] Lu JJY, Chen JY, Hsu TY, Yu WCY, Su IJ, Yang CS (1997). Cooperative interaction between Bcl-2 and Epstein-Barr virus latent membrane protein 1 in the growth transformation of human epithelial cells. J Gen Virol.

[18] Murray P, Swinnen L, Constandinou C, Pyle J, Carr T, Hardwick M, Ambinder RF (1996). BCL-2 but not its Epstein-Barr Virus-encoded homologue, BHRF1, is commonly expressed in Posttransplantation Lymphoproliferative Disorders. Blood.

[19] Kvansakul M, Wei AH, Fletcher K, Willis SN, Chen L (2010). Structural basis for apoptosis inhibition by Epstein-Barr virus BHRF1. PLoS Pathog.

[20] Bellows DS, Howell M, Pearson C, Hazlewood SA, Hardwick JM (2002). Epstein-Barr virus BALF1 is a BCL-2-like antagonist of the herpesvirus antiapoptotic BCL-2 proteins. J Virol.

[21] Marshall WL, Yim C, Gustafson E, Graf T, Sage DR, Hanify K, Williams L, Fingeroth J, Finberg RW (1999). Epstein-Barr virus encodes a novel homolog of the bcl-2 oncogene that inhibits apoptosis and associates with Bax and Bak. J Virol.

[22] Alibek K, Baiken Y, Kakpenova A, Mussabekova A, Zhussupbekova S, Akan M, Sultankulov B (2014). Implication of human herpesviruses in oncogenesis through immune evasion and suppression. Infect Agents Canc.

[23] Sinclair J, Sissons P (2006). Latency and reactivation of human cytomegalovirus. J Gen Virol.

[24] Andoniou CE, Degli-Esposti MA (2006). Insights into the mechanisms of CMV-mediated interference with cellular apoptosis. Immunol Cell Biol.

[25] Zhang A, Hildreth RL, Colberg-Poley AM (2013). Human cytomegalovirus inhibits apoptosis by proteasome-mediated degradation of bax at endoplasmic reticulum-mitochondrion contacts. J Virol.

[26] McCormick AL (2008). Control of apoptosis by human cytomegalovirus. Hum Cytomegalovirus.

[27] Fedorova NE, Sokolova TM, Medzhidova MG, Kushch AA (2010). Different regulation of mitochondrial apoptosis and Bcl-2 gene expression in quescent and proliferative human fibroblasts infected with cytomegalovirus. Tsitologiia.

[28] Lares AP, Tu CC, Spencer JV (2013). The human cytomegalovirus US27 gene product enhances cell proliferation and alters cellular gene expression. Virus Res.

[29] Wang X, Belguise K, Kersual N, Kirsch KH, Mineva ND, Galtier F, Chalbos D, Sonenshein GE (2007). Oestrogen signalling inhibits invasive phenotype by repressing RelB and its target BCL2. Nat Cell Biol.

[30] Khan KA, Coaquette A, Davrinche C, Herbein G (2009). Bcl-3-regulated transcription from major immediate-early promoter of human cytomegalovirus in monocyte-derived macrophages. J Immunol.

[31] Chang Y, Cesarman E, Pessin MS, Lee F, Culpepper J, Knowles DM, Moore PS (1994). Identification of herpesvirus-like DNA sequences in AIDS-associated Kaposi’s sarcoma. Science.

[32] Cesarman E, Chang Y, Moore PS, Said JW, Knowles DM (1995). Kaposi’s sarcoma-associated herpesvirus-like DNA sequences in AIDS-related body-cavity-based lymphomas. N Engl J Med.

[33] Corbellino M, Poirel L, Aubin JT, Paulli M, Magrini U, Bestetti G, Galli M, Parravicini C (1996). The role of human herpesvirus 8 and Epstein-Barr virus in the pathogenesis of giant lymph node hyperplasia (Castleman’s disease). Clin Infect Dis.

[34] Russo JJ, Bohenzky RA, Chien MC, Chen J, Yan M, Maddalena D, Parry JP, Peruzzi D, Edelman IS, Chang Y, Moore PS (1996). Nucleotide sequence of the Kaposi sarcoma associated herpesvirus (HHV8). Proc Natl Acad Sci USA.

[35] Moore PS, Chang Y (2001). Molecular virology of Kaposi’s sarcoma-associated herpesvirus. Philos Trans R Soc Lond B Biol Sci.

[36] West JT, Wood C (2003). The role of Kaposi’s sarcoma-associated herpesvirus/human herpesvirus-8 regulator of transcription activation (RTA) in control of gene expression. Oncogene.

[37] Montaner S, Sodhi A, Molinolo A, Bugge TH, Sawai ET, He Y, Li Y, Ray PE, Gutkind JS (2003). Endothelial infection with KSHV genes in vivo reveals that vGPCR initiates Kaposi’s sarcomagenesis and can promote the tumorigenic potential of viral latent genes. Cancer Cell.

[38] Huang Q, Petros AM, Virgin HW, Fesik SW, Olejniczak ET (2002). Solution structure of a Bcl-2 homolog from Kaposi sarcoma virus. Proc Natl Acad Sci USA.

[39] Sattler M, Liang H, Nettesheim D, Meadows RP, Harlan JE, Eberstadt M, Yoon HS, Shuker SB, Chang BS, Minn AJ, Thompson CB, Fesik SW (1997). Structure of Bcl-xL-Bak peptide complex: recognition between regulators of apoptosis. Science.

[40] Suzuki M, Youle RJ, Tjandra N (2000). Structure of Bax: coregulation of dimer formation and intracellular localization. Cell.

[41] Loh J, Huang Q, Petros AM, Nettesheim D, van Dyk LF, Labrada L, Speck SH, Levine B, Olejniczak ET, Virgin HW (2005). A surface groove essential for viral Bcl-2 function during chronic infection in vivo. PLoS Pathog.

[42] Wei Y, Pattingre S, Sinha S, Bassik M, Levine B (2008). JNK1-mediated phosphorylation of Bcl 2 regulates starvation-induced autophagy. Mol Cell.

[43] Dourmishev LA, Dourmishev AL, Palmeri D, Schwartz RA, Lukac DM (2003). Molecular genetics of Kaposi’s sarcoma-associated herpesvirus (human herpesvirus-8) epidemiology and pathogenesis. Microbiol Mol Biol Rev.

[44] Cuconati A, White E (2002). Viral homologs of BCL-2: role of apoptosis in the regulation of virus infection. Genes Dev.

[45] Cheng EH-Y, Nicholas J, Bellows DS, Hayward GS, Guo H-G, Reitz MS, Hardwick JM (1997). A Bcl-2 homolog encoded by Kaposi sarcoma-associated virus, human herpesvirus 8, inhibits apoptosis but does not heterodimerize with Bax or Bak. Proc Natl Acad Sci USA.

[46] Sarid R, Sata T, Bohenzky RA, Russo JJ, Chang Y (1997). Kaposi’s sarcoma-associated herpesvirus encodes a functional bcl-2 homologue. Nat Med.

[47] Widmer I, Wernli M, Bachmann F, Gudat F, Cathomas G, Erb P (2002). Differential expression of viral Bcl 2 encoded by Kaposi’s sarcoma-associated herpesvirus and human Bcl-2 in primary effusion lymphoma cells and Kaposi’s sarcoma lesions. J Virol.

[48] Ojala PM, Yamamoto K, Castanos-Velez E, Biberfeld P, Korsmeyer SJ, Makela TP (2000). The apoptotic v-cyclin-CDK6 complex phosphorylates and inactivates Bcl-2. Nat Cell Biol.

[49] Bellows DS, Chau BN, Lee P, Lazebnik Y, Burns WH, Hardwick JM (2000). Antiapoptotic herpesvirus Bcl-2 homologs escape caspase-mediated conversion to proapoptotic proteins. J Virol.

[50] Walensky LD (2006). BCL-2 in the crosshairs: tipping the balance of life and death. Cell Death Differ.

[51] Kalt I, Borodianskiy-Shteinberg T, Schachor A, Sarid R (2010). GLTSCR2/PICT-1, a putative tumor suppressor gene product, induces the nucleolar targeting of the Kaposi’s Sarcoma Associated Herpesvirus KS-Bcl-2 protein. J Virol.

[52] Bernard HU (2005). The clinical importance of the nomenclature, evolution and taxonomy of human papillomaviruses. J Clin Virol.

[53] Bosch FX, Lorincz A, Munoz N, Meijer CJ, Shah KV (2002). The causal relation between human papillomavirus and cervical cancer. J Clin Pathol.

[54] Harwood CA, Surentheran T, Sasieni P, Proby CM, Bordea C, Leigh IM, Wojnarowska F, Breuer J, McGregor JM (2004). Increased risk of skin cancer associated with the presence of epidermodysplasia verruciformis human papillomavirus types in normal skin. Br J Dermatol.

[55] Doorbar J, Foo C, Coleman N, Medcalf E, Hartley O, Prospero T, Napthine S, Sterling J, Winter G, Griffin H (1997). Characterisation of events during the late stages of HPV16 infection in vivo using high affinity synthetic fabs to E4. Virology.

[56] Culp TD, Christensen N (2004). Kinetics of in vitro adsorption and entry of papillomavirus virions. Virology.

[57] Wilson VG, West M, Woytek K, Rangasamy D (2002). Papillomavirus E1 proteins: form, function, and features. Virus Genes.

[58] Munger K, Basile JR, Duensing S, Eichten A, Gonzalez SL, Grace M, Zacny VL (2001). Biological activities and molecular targets of the human papillomavirus E7 oncoprotein. Oncogene.

[59] Lee JO, Russo AA, Pavletich NP (1998). Structure of the retinoblastoma tumour-suppressor pocket domain bound to a peptide from HPV E7. Nature.

[60] Longworth MS, Laimins LA (2004). The binding of histone deacetylases and the integrity of zinc finger-like motifs of the E7 protein are essential for the life cycle of human papillomavirus type 31. J Virol.

[61] Funk JO, Waga S, Harry JB, Espling E, Stillman B, Galloway DA (1997). Inhibition of CDK activity and PCNA-dependent DNA replication by p21 is blocked by interaction with the HPV16 E7 oncoprotein. Genes Dev.

[62] Scheffner M, Werness BA, Huibregtse JM, Levine AJ, Howley PM (1990). The E6 oncoprotein encoded by human papillomavirus types 16 and 18 promotes the degradation of p53. Cell.

[63] Liang XH, Mungal S, Ayscue A, Meissner JD, Wodnicki P, Hockenbery D, Lockett S, Herman B (1995). Bcl-2 protooncogene expression in cervical carcinoma cell lines containing inactive p53. J Cell Biochem.

[64] Nguyen MM, Nguyen ML, Caruana G, Bernstein A, Lambert PF, Griep AE (2003). Requirement of PDZ-containing proteins for cell cycle regulation and differentiation in the mouse lens epithelium. Mol Cell Biol.

[65] Middleton K, Peh W, Southern SA, Griffin HM, Sotlar K, Nakahara T, El-Sherif A (2003). Organisation of the human papillomavirus productive cycle during neoplastic progression provides a basis for the selection of diagnostic markers. J Virol.

[66] Leechanachai P, Banks L, Moreau F, Matlashewski G (1992). The E5 gene from human papillomavirus type 16 is an oncogene which enhances growth factor-mediated signal transduction to the nucleus. Oncogene.

[67] Straight SW, Hinkle PM, Jewers RJ, McCance DJ (1993). The E5 oncoprotein of human papillomavirus type 16 transforms fibroblasts and effects the downregulation of the epidermal growth factor receptor in keratinocytes. J Virol.

[68] Williams SMG, Disbrow GL, Schlegel R, Lee D, Threadgill DW, Lambert PF (2005). Requirement of epidermal growth factor receptor for hyperplasia induced by E5, a high-risk human papillomavirus oncogene. Cancer Res.

[69] Maufort JP, Williams SMG, Pitot HC, Lambert PF (2007). Human papillomavirus 16 E5 oncogene contributes to two stages of skin carcinogenesis. Cancer Res.

[70] Valle GF, Banks L (1995). The human papillomavirus (HPV)-6 and HPV-16 E5 proteins cooperate with HPV-16 E7 in the transformation of primary rodent cells. J Gen Virol.

[71] Oh JM, Kim SH, Cho EA, Song YS, Kim WH, Juhnn YS (2010). Human papillomavirus type 16 E5 protein inhibits hydrogen peroxide-induced apoptosis by stimulating ubiquitin proteasome mediated degradation of Bax in human cervical cancer cells. Carcinogenesis.

[72] Mesri EA, Feitelson MA, Munger K (2014). Human viral oncogenesis: a cancer hallmarks analysis. Cell Host Microbe.

[73] Wongstaal F, Gallo RC (1985). Human t-lymphotropic retroviruses. Nature.

[74] Simonis N, Rual J-F, Lemmens I, Boxus M, Hirozane-Kishikawa T, Gatot J-S, Dricot A, Hao T, Vertommen D, Legros S, Daakour S, Klitgord N, Martin M, Willaert J-F, Dequiedt F, Navratil V, Cusick ME, Burny A, Van Lint C, Hill DE, Tevernier J, Kettmann R, Vidal M, Twizere J-C (2012). Host pathogen interactome mapping for HTLV-1 and −2 retroviruses. Retrovirology.

[75] Chlichlia K, Khazaie K (2010). HTLV-1 Tax: Linking transformation, DNA damage and apoptotic T-cell death. Chem Biol Interact.

[76] Harakeh S, Diab-Assaf M, Khalife JC, Abu-El-Ardat KA, Baydoun E, Niedzwiecki A, El-Sabban ME, Rath M (2007). Ascorbic acid induces apoptosis in adult T-cell leukemia. Anticancer Res.

[77] Azran I, Schavinsky-Khrapunsky Y, Aboud M (2004). Role of Tax protein in human T-cell leukemia virus type-I leukemogenicity. Retrovirology.

[78] Taylor JM, Nicot C (2008). HTLV-1 and apoptosis: role in cellular transformation and recent advances in therapeutic approaches. Apoptosis.

[79] Gatza ML, Watt JC, Marriott SJ (2003). Cellular transformation by the HTLV-I Tax protein, a jack-of-all-trades. Oncogene.

[80] Bogenberger JM, Laybourn PJ (2008). Human T lymphotropic virus type 1 protein tax reduces histone levels. Retrovirology.

[81] D’Agostino DM, Bernardi P, Chieco-Bianchi L, Ciminale V (2005). Mitochondria as functional targets of proteins coded by human tumor viruses. Adv Cancer Res.

[82] Grassmann R, Aboud M, Jeang KT (2005). Molecular mechanisms of cellular transformation by HTLV-1 Tax. Oncogene.

[83] Ishikawa C, Nakachi S, Senba M, Sugai M, Mori N (2011). Activation of AID by human T-cell leukemia virus Tax oncoprotein and the possible role of its constitutive expression in ATL genesis. Carcinogenesis.

[84] Matsuoka MM, Jeang KT (2007). Human T-cell leukaemia virus type 1 (HTLV-1) infectivity and cellular transformation. Nat Rev Cancer.

[85] Macaire H, Riquet A, Moncollin V, Biemont-Trescol MC, Dodon MD, Hermine O, Debaud AL, Mahieux R, Mesnard JM, Pierre M, Gazzolo L, Bonnefoy N, Valentin H (2012). Tax protein-induced expression of antiapoptotic Bfl-1 protein contributes to survival of human T-cell leukemia virus type 1 (HTLV-1)-infected T-cells. J Biol Chem.

[86] Nakashima K, Kawakami A, Hida A, Yamasaki S, Nakamura H, Kamachi M, Miyashita T, Tanaka F, Izumi Y, Tamai M, Ida H, Furuyama M, Koji T, Nakamura T, Migita K, Origuchi T, Eguchi K (2003). Protection of mitochondrial perturbation by human T-lymphotropic virus type 1 tax through induction of Bcl-xL expression. J Lab Clin Med.

[87] Qu Z, Xiao G (2011). Human T-Cell lymphotropic virus: a model of NF-kappa B-associated tumorigenesis. Viruses-Basel.

[88] Nicot C, Mahieux R, Takemoto S, Franchini G (2000). Bcl-X-L is up-regulated by HTLV-I and HTLV-II in vitro and in ex vivo ATLL samples. Blood.

[89] Cook LB, Elemans M, Rowan AG, Asquith B (2013). HTLV-1: persistence and pathogenesis. Virology.

[90] Ishitsuka K, Kunami N, Katsuya H, Nogami R, Ishikawa C, Yotsumoto F, Tanji H, Mori N, Takeshita M, Miyamoto S, Tamura K (2012). Targeting Bcl-2 family proteins in adult T-cell leukemia/lymphoma: in vitro and in vivo effects of the novel Bcl-2 family inhibitor ABT-737. Cancer Lett.

[91] Krueger A, Fas SC, Giaisi M, Bleumink M, Merling A, Stumpf C, Baumann S, Holtkotte D, Bosch V, Krammer PH, Li-Weber M (2006). HTLV-1 tax protects against CD95-mediated apoptosis by induction of the cellular FLICE-inhibitory protein (c-FLIP). Blood.

[92] Brauweiler A, Garrus JE, Reed JC, Nyborg JK (1997). Repression of Bax gene expression by the HTLV-I tax protein: implications for suppression of apoptosis in virally infected cells. Virology.

[93] Reed JC, Zha HB, AimeSempe C, Takayama S, Wang HG (1996). Structure-function analysis of bcl-2 family proteins - Regulators of programmed cell death. Mechanisms of Lymphocyte Activation and Immune Regulation.

[94] Nair A, Michael B, Hiraragi H, Fernandez S, Feuer G, Boris-Lawrie K, Lairmore M (2005). Human T lymphotropic virus type 1 accessory protein p12(I) modulates calcium-mediated cellular gene expression and enhances p300 expression in T lymphocytes. Aids Res Hum Retrovir.

[95] Li Y, Zhang Q, Liu Y, Luo Z, Kang L, Qu J, Liu W, Xia X, Liu Y, Wu K, Wu J (2012). Hepatitis C virus activates Bcl-2 and MMP-2 expression through multiple cellular signalling pathways. J Virol.

[96] Alenzi FQ, El-Nashar EM, Al-Ghamdi SS, Abbas MY, Hamad AM, El-Saeed OM, Wyse RKH, Lofty M (2010). Investigation of Bcl-2 and PCNA in hepatocellular carcinoma: relation to chronic HCV. J Egypt Natl Canc Inst.

[97] Fabregat I (2009). Dysregulation of apoptosis in hepatocellular carcinoma cells. World J Gastroenterol.

[98] Zekri ARN, Bahnassy AA, Hafez MM, Hassan ZK, Kamel M, Loufty SA, Sherif GM, El Zayadi AR, Daoud SS (2011). Characterization of chronic HCV infection-induced apoptosis. Comp Hepatol.

[99] Houghton M, Weiner A, Han J, Kuo G, Choo QL (1991). Molecular biology of the hepatitis C viruses: implications for diagnosis, development and control of viral disease. Hepatology.

[100] Yoshida T, Hanada T, Tokuhisa T, Kosai K, Sata M, Kohara M, Yoshimura A (2002). Activation of STAT3 by the hepatitis C virus core protein leads to cellular transformation. J Exp Med.

[101] Otsuka M, Kato N, Taniguchi H, Yoshida H, Goto T, Shiratori Y, Omata M (2002). Hepatitis C virus core protein inhibits apoptosis via enhanced Bcl-xL expression. Virology.

[102] Chou AH, Tsai HF, Wu YY, Hu CY, Hwang LH, Hsu PI, Hsu PN (2005). Hepatitis C virus core protein modulates TRAIL-mediated apoptosis by enhancing bid cleavage and activation of mitochondria apoptosis signaling pathway. J Immunol.

[103] Gao L, Aizaki H, He JW, Lai MM (2004). Interactions between viral non-structural proteins and host protein hVAP-33 mediate the formation of hepatitis C virus RNA replication complex on lipid raft. J Virol.

[104] Gong G, Waris G, Tanveer R, Siddiqui A (2001). Human hepatitis C virus NS5A protein alters intracellular calcium levels, induces oxidative stress, and activates STAT-3 and NF-kappa B. Proc Natl Acad Sci U S A.

[105] Arima N, Kao CY, Licht T, Padmanabhan R, Sasaguri Y, Padmanabhan R (2001). Modulation of cell growth by the hepatitis C virus nonstructural protein NS5A. J Biol Chem.

[106] Lan KH, Sheu ML, Hwang SJ, Yen SH, Chen SY, Wu JC, Wang YJ, Kato N, Omata M, Chang FY, Lee SD (2002). HCV NS5A interacts with p53 and inhibits p53-mediated Apoptosis. Oncogene.

[107] Korsmeyer SJ (1995). Regulators of cell death. Trends Genet.

[108] Wang J, Tong W, Zhang X, Chen L, Yi Z, Pan T, Hu Y, Xiang L, Yuan Z (2006). Hepatitis C virus non-structural protein NS5A interacts with FKBP38 and inhibits apoptosis in Huh7 hepatoma cells. FEBS Lett.

[109] Chung YL, Sheu ML, Yen SH (2003). Hepatitis C virus NS5A as a potential viral Bcl-2 homologue interacts with Bax and inhibits apoptosis in hepatocellular carcinoma. Int J Cancer.

[110] Strasser A, Whittingham S, Vaux DL, Bath ML, Adams JM, Cory S, Harris AW (1991). Enforced bcl2 expression in b-lymphoid cells prolongs antibody-responses and elicits autoimmune-disease. Proc Natl Acad Sci U S A.

[111] Sentman CL, Shutter JR, Hockenbery D, Kanagawa O, Korsmeyer SJ (1991). Bcl-2 inhibits multiple forms of apoptosis but not negative selection in THYMOCYTES. Cell.

[112] Schmitt CA, Rosenthal CT, Lowe SW (2000). Genetic analysis of chemoresistance in primary murine lymphomas. Nat Med.

[113] Amundson SA, Myers TG, Scudiero D, Kitada S, Reed JC, Fornace AJ (2000). An informatics approach identifying markers of chemosensitivity in human cancer cell lines. Cancer Res.

[114] Middleton T, Gahn TA, Martin JM, Sugden B (1991). Immortalizing genes of Epstein-Barr-virus. Adv Virus Res.

[115] Henderson S, Rowe M, Gregory C, Croomcarter D, Wang F, Longnecker R, Kieff E, Rickinson A (1991). Induction of bcl-2 expression by Epstein-Barr-virus latent membrane protein-1 protects infected B-cells from programmed cell-death. Cell.

[116] Kenney JL, Guinness ME, Curiel F, Lacy J (1998). Antisense to the Epstein-Barr virus (EBV)-encoded latent membrane protein 1 (LMP-1) suppresses LMP-1 and Bcl-2 expression and promotes apoptosis in EBV-immortalized B cells. Blood.

[117] Guinness ME, Kenney JL, Reiss M, Lacy J (2000). Bcl-2 antisense oligodeoxynucleotide therapy of Epstein-Barr virus-associated lymphoproliferative disease in severe combined immunodeficient mice. Cancer Res.

[118] Johnsen JI, Baryawno N, Soderberg-Naucler C (2011). Is human cytomegalovirus a target in cancer therapy?. Oncotarget.

[119] Biron KK (2006). Antiviral drugs for cytomegalovirus diseases. Antiviral Res.

[120] Schreiber A, Haerter G, Schubert A, Bunjes D, Mertens T, Michel D (2009). Antiviral treatment of cytomegalovirus infection and resistant strains. Expert Opin Pharmacother.

[121] Dziurzynski K, Wei J, Qiao W, Hatiboglu MA, Kong L-Y, Wu A, Wang Y, Cahill D, Levine N, Prabhu S, Rao G, Sawaya R, Heimberger AB (2011). Glioma-associated cytomegalovirus mediates subversion of the monocyte lineage to a tumor propagating phenotype. Clin Cancer Res.

[122] Sampson JH, Mitchell DA (2011). Is cytomegalovirus a therapeutic target in glioblastoma?. Clin Cancer Res.

[123] Wen KW, Damania B (2010). Kaposi sarcoma-associated herpesvirus (KSHV): molecular biology and oncogenesis. Cancer Lett.

[124] Sarek G, Kurki S, Enback J, Iotzova G, Haas J, Laakkonen P, Laiho M, Ojala PM (2007). Reactivation of the p53 pathway as a treatment modality for KSHV-induced lymphomas. J Clin Investig.

[125] Godfrey A, Anderson J, Papanastasiou A, Takeuchi Y, Boshoff C (2005). Inhibiting primary effusion lymphoma, by lentiviral vectors encoding short hairpin RNA. Blood.

[126] Stallone G, Schena A, Infante B, DiPaolo S, Loverre A, Maggio G, Ranieri E, Gesualdo L, Schena FP, Grandaliano G (2005). Sirolimus for kaposi’s sarcoma in renal – transplant recipients. N Engl J Med.

[127] Jiang M, Milner J (2002). Selective silencing of viral gene expression in HPV-positive human cervical carcinoma cells treated with siRNA, a primer of RNA interference. Oncogene.

[128] Kjaer SK, Sigurdsson K, Iversen OE, Hernandez-Avila M, Wheeler CM, Perez G, Haupt RM, Brown DR, Koutsky LA, Tay EH, García P, Ault KA, Garland SM, Leodolter S, Olsson SE, Tang GW, Ferris DG, Paavonen J, Lehtinen M, Steben M, Bosch FX, Dillner J, Joura EA, Majewski S, Muñoz N, Myers ER, Villa LL, Taddeo FJ, Roberts C, Tadesse A (2009). A pooled analysis of continued prophylactic efficacy of quadrivalent human papillomavirus (Types 6/11/16/18) vaccine against high-grade cervical and external genital lesions. Canc Prev Res.

[129] Paavonen J, Naud P, Salmeron J, Wheeler CM, Chow SN, Apter D, Kitchener H, Castellsague X, Teixeira JC, Skinner SR, Hedrick J, Jaisamrarn U, Limson G, Garland S, Szarewski A, Romanowski B, Aoki FY, Schwarz TF, Poppe WA, Bosch FX, Jenkins D, Hardt K, Zahaf T, Descamps D, Struyf F, Lehtinen M, Dubin G, HPV PATRICIA Study Group (2009). Efficacy of human papillomavirus (HPV)-16/18 AS04-adjuvanted vaccine against cervical infection and precancer caused by oncogenic HPV types (PATRICIA): final analysis of a double-blind, randomised study in young women. Lancet.

[130] Pang CL, Thierry F (2013). Human papillomavirus proteins as prospective therapeutic targets. Microb Pathog.

[131] Liu DW, Tsao YP, Hsieh CH, Hsieh JT, Kung JT, Chiang CL, Chen SL (2000). Induction of CD8 T cells by vaccination with recombinant adenovirus expressing human papillomavirus type 16 E5 gene reduces tumor growth. J Virol.

[132] Kfoury Y, Nasr R, Hermine O, de The H, Bazarbachi A (2005). Proapoptotic regimes for HTLV-I-transformed cells: targeting Tax and the NF-kB pathway. Cell Death Differ.

[133] Nasr R, El Hajj H, Kfoury Y, de Thé H, Hermine O, Bazarbachi A (2011). Controversies in targeted therapy of adult T cell leukemia/lymphoma: ON target or OFF target effects?. Viruses.

[134] Dewan MZ, Uchihara J, Terashima K, Honda M, Sata T, Ito M, Fujii N, Uozumi K, Tsukasaki K, Tomonaga M, Kubuki Y, Okayama A, Toi M, Mori N, Yamamoto N (2006). Efficient intervention of growth and infiltration of primary adult T-cell leukemia cells by an HIV protease inhibitor, ritonavir. Blood.

[135] Ikezoe T, Yang Y, Bandobashi K, Saito T, Takemoto S, Machida H, Togitani K, Koeffler HP, Taguchi H (2005). Oridonin, a diterpenoid purified from Rabdosia rubescens, inhibits the proliferation of cells from lymphoid malignancies in association with blockade of the NF-kappa B signal pathways. Mol Cancer Ther.

[136] Zhang J, Nagasaki M, Tanaka Y, Morikawa S (2003). Capsaicin inhibits growth of adult T-cell leukemia cells. Leuk Res.

[137] Moarbess G, El-Hajj H, Kfoury Y, El-Sabban ME, Lepelletier Y, Hermine O, Deleuze-Masquéfa C, Bonnet PA, Bazarbachi A (2008). EAPB0203, a member of the imidazoquinoxaline family, inhibits growth and induces caspase-dependent apoptosis in T-cell lymphomas and HTLV-I-associated adult T-cell leukemia/lymphoma. Blood.

[138] Fischer R, Baumert T, Blum HE (2007). Hepatitis C virus infection and apoptosis. World J Gastroenterol.

[139] Poynard T, Marcellin P, Lee SS, Niederau C, Minuk GS, Ideo G, Bain V, Heathcote J, Zeuzem S, Trepo C, Albrecht J (1998). Randomised trial of interferon α2b plus ribavirin for 48 weeks or for 24 weeks versus interferon α2b plus placebo for 48 weeks for treatment of chronic infection with hepatitis C virus. International Hepatitis Interventional Therapy Group (IHIT). Lancet.

[140] Lawitz EJ, Rodriguez-Torres M, Muir A, Keane J, Kieffer T, McNair L, McHutchinson JG (2006). 28 Days of the hepatitis C protease inhibitor VX-950, in combination with PEG Interferon-α-2a and ribavirin, is well-tolerated and demonstrates robust antiviral effects. Gastroenterology.

[141] Cholongitas E, Papatheodoridis GV (2014). Sofosbuvir: a novel oral agent for chronic hepatitis C. Ann Gastroenterol.

[142] Pawlotsky JM, McHutchison JG: Hepatitis C (2004). Development of new drugs and clinical trials: promises and pitfalls. Summary of an AASLD hepatitis single topic conference, Chicago, IL, February 27-March 1, 2003. Hepatology.

[143] Soriano V, Peters MG, Zeuzem S (2009). New therapies for hepatitis C virus infection. Clin Infect Dis.

